# Long Non-Coding RNAs: Bridging Cancer-Associated Thrombosis and Clinical Outcome of Ovarian Cancer Patients

**DOI:** 10.3390/ijms25010140

**Published:** 2023-12-21

**Authors:** Inês Soares Marques, Valéria Tavares, Joana Savva-Bordalo, Mariana Rei, Joana Liz-Pimenta, Inês Guerra de Melo, Joana Assis, Deolinda Pereira, Rui Medeiros

**Affiliations:** 1 Molecular Oncology and Viral Pathology Group, Research Center of IPO Porto (CI-IPOP)/Pathology and Laboratory Medicine Department, Clinical Pathology SV/RISE@CI-IPOP (Health Research Network), Portuguese Oncology Institute of Porto (IPO Porto)/Porto Comprehensive Cancer Centre (Porto.CCC), 4200-072 Porto, Portugal; ines.soares@ipoporto.min-saude.pt (I.S.M.); valeria.tavares@ipoporto.min-saude.pt (V.T.); ines.melo@ipoporto.min-saude.pt (I.G.d.M.); 2 Faculty of Sciences of the University of Porto (FCUP), 4169-007 Porto, Portugal; 3 Faculty of Medicine of the University of Porto (FMUP), 4200-072 Porto, Portugal; jpimenta@chtmad.min-saude.pt; 4 Abel Salazar Institute for the Biomedical Sciences (ICBAS), University of Porto, 4050-313 Porto, Portugal; 5 Department of Medical Oncology, Portuguese Institute of Oncology of Porto (IPO Porto), 4200-072 Porto, Portugal; joana.sa@ipoporto.min-saude.pt (J.S.-B.); dpereira@ipoporto.min-saude.pt (D.P.); 6 Department of Gynaecology, Portuguese Institute of Oncology of Porto (IPO Porto), 4200-072 Porto, Portugal; marianacruzrei@gmail.com; 7 Department of Medical Oncology, Centro Hospitalar de Trás-os-Montes e Alto Douro (CHTMAD), 5000-508 Vila Real, Portugal; 8 Clinical Research Unit, Research Center of IPO Porto (CI-IPOP)/RISE@CI-IPOP (Health Research Network), Portuguese Oncology Institute of Porto (IPO Porto)/Porto Comprehensive Cancer Center (Porto.CCC), 4200-072 Porto, Portugal; joana.assis@ipoporto.min-saude.pt; 9 Faculty of Health Sciences, Fernando Pessoa University, 4200-150 Porto, Portugal; 10 Research Department, Portuguese League Against Cancer (NRNorte), 4200-172 Porto, Portugal

**Keywords:** epithelial ovarian carcinoma, venous thromboembolism, RNA, long non-coding, prognosis, thromboprophylaxis

## Abstract

Ovarian cancer (OC) and venous thromboembolism (VTE) have a close relationship, in which tumour cells surpass the haemostatic system to drive cancer progression. Long non-coding RNAs (lncRNAs) have been implicated in VTE pathogenesis, yet their roles in cancer-associated thrombosis (CAT) and their prognostic value are unexplored. Understanding how these lncRNAs influence venous thrombogenesis and ovarian tumorigenesis may lead to the identification of valuable biomarkers for VTE and OC management. Thus, this study evaluated the impact of five lncRNAs, namely MALAT1, TUG1, NEAT1, XIST and MEG8, on a cohort of 40 OC patients. Patients who developed VTE after OC diagnosis had worse overall survival compared to their counterparts (log-rank test, *p* = 0.028). Elevated pre-chemotherapy MEG8 levels in peripheral blood cells (PBCs) predicted VTE after OC diagnosis (Mann–Whitney *U* test, *p* = 0.037; *Χ*^2^ test, *p* = 0.033). In opposition, its low levels were linked to a higher risk of OC progression (adjusted hazard ratio (aHR) = 3.00; *p* = 0.039). Furthermore, low pre-chemotherapy NEAT1 levels in PBCs were associated with a higher risk of death (aHR = 6.25; *p* = 0.008). As for the remaining lncRNAs, no significant association with VTE incidence, OC progression or related mortality was observed. Future investigation with external validation in larger cohorts is needed to dissect the implications of the evaluated lncRNAs in OC patients.

## 1. Introduction

Regarded as a silent killer, OC is often diagnosed at advanced stages and patients frequently develop therapy resistance [1,2,3]. These two factors contribute to a high rate of disease recurrence and a five-year survival rate lower than 50%, making OC the most lethal gynaecological tumour [1]. Therefore, more suitable prognostic biomarkers are required to personalise the disease management, which demands a better knowledge of ovarian tumorigenesis [4].

Although most studies on OC are focused on tumour cells, understanding the surrounding microenvironment and how tumour cells and stroma interact to promote disease aggressiveness is critical [5]. For instance, key players of the haemostatic system in the tumour microenvironment are known to establish a bidirectional relationship with cancer [6,7]. Specifically, tumour cells can activate the coagulation cascade and platelets and inhibit fibrinolysis through the secretion of several factors, alone or within microparticles, namely tissue factor (TF), podoplanin and plasminogen activation inhibitor-1 (PAI-1), respectively [8]. In addition, cytokines derived from tumour cells can lead to the recruitment and activation of leucocytes, such as monocytes and neutrophils. Activated monocytes can release cancer procoagulant (CP) and TF-positive microparticles, while neutrophil extracellular traps (NETs) can capture and activate platelets, favouring the buildup of fibrin. In this process called immunothrombosis, platelets, leukocytes and endothelial cells interact, augmenting thrombogenesis [9,10,11,12]. On the other hand, haemostatic components highjacked by tumour cells can fuel their progression, for instance, by inducing tumour neoangiogenesis [8]. As a result, this complex network increases the susceptibility of oncological patients to thrombotic events while promoting tumour aggressiveness [13,14].

Venous thromboembolism (VTE) is a cardiovascular disease encompassing deep venous thrombosis (DVT) and pulmonary embolism (PE). This life-threatening condition, which results from a haemostatic imbalance, is common, particularly in regions with high Human Development Index (HDI) levels [4]. Cancer patients are four to seven times more likely to develop VTE than those without malignancy, with approximately 15% of the patients experiencing a VTE episode [15]. Conversely, the risk for cancer diagnosis is higher at least 2 years after the first episode of idiopathic VTE [16]. The disease is also associated with a poor prognosis, being the second-leading cause of death among oncological patients [8]. As such, there has been a growing effort to find predictive markers for cancer-associated thrombosis (CAT).

VTE occurrence is commonly observed among OC patients, with an incidence ranging from 5 to 20%, which increases their mortality rates [4]. Given the roles of the tumour coagulome in cancer growth and progression, it is important to identify biomarkers of OC-related VTE as they might improve the management of both VTE and OC [17]. Recently, multiple long non-coding RNAs (lncRNAs) were shown to influence venous thrombus formation and resolution; however, the data specifically among cancer patients are scarce [18]. Given the impact of platelets, endothelial cells and leukocytes on both thrombogenesis and tumour progression, as well as the high incidence and negative effect of VTE among OC patients, VTE-associated lncRNAs may influence the pathogenesis of OC-related VTE and constitute valuable OC prognostic biomarkers regardless of VTE. Thus, this study evaluated the expression of lncRNAs in peripheral blood cells (PBCs) and its implications in CAT and the prognosis of OC patients.

## 2. Results

### 2.1. Implications of VTE on Patients’ Prognosis

Among 30 patients with sufficient follow-up time, seven (23.3%) developed OC-related VTE, including two patients with VTE before and five after OC diagnosis. Regarding the type of VTE, five events (71.4%) were DVT and two (28.6%) were PE. Most of the events (4 (57.1%)) were symptomatic. Among the patients with VTE before cancer diagnosis, the mean interval between VTE and OC diagnosis was 3.5 ± 2.1 months. As for those with VTE after cancer diagnosis, the mean time to VTE was 9.2 ± 8.9 months.

The Khorana score (KS) did not present a significant predictive impact on VTE incidence among the OC patients (≥2 versus (vs.) <2; *Χ*^2^, *p* > 0.05). Also, no significant association between VTE development and patients’ demographical and clinicopathological features, baseline full blood count and coagulation tests was observed (*Χ*^2^, *p* > 0.05).

**Figure 1:**
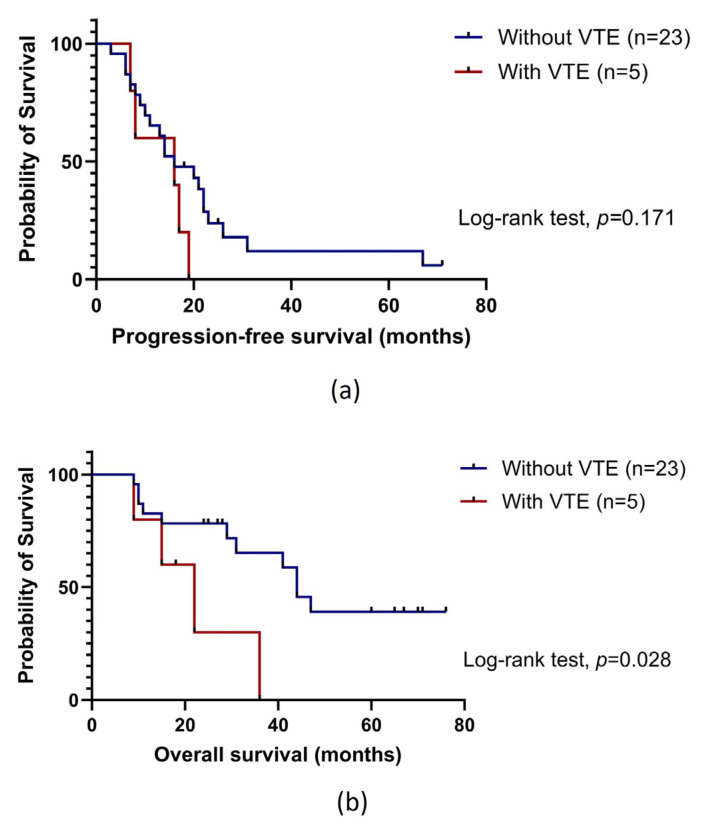
Progression-free survival (**a**) and overall survival (**b**) by Kaplan–Meier and log-rank test for ovarian cancer (OC) patients (n = 28), according to venous thromboembolism (VTE) occurrence after cancer diagnosis. (**a**) No association between VTE and patients’ progression-free survival (PFS) was observed among those with the condition after cancer diagnosis (log-rank test, *p* = 0.171). (**b**) Patients with VTE after OC diagnosis had lower overall survival (OS) compared to their counterparts (mean OS of 22.2 ± 5.6 months and 47.5 months ± 5.9 months, respectively; log-rank test, *p* = 0.028).

No association between VTE and patients’ progression-free survival (PFS) was detected (log-rank test, *p* = 0.171, Figure 1a). Contrariwise, those with VTE had a worse overall survival (OS) than their counterparts (mean OS of 26.5 ± 5.2 months and 47.5 months ± 5.9 months, respectively; log-rank test, *p* = 0.044). This negative effect was even more evident when focusing on those with VTE after OC diagnosis (mean OS of 22.2 ± 5.6 months and 47.5 months ± 5.9 months, respectively; log-rank test, *p* = 0.028, Figure 1b).

### 2.2. LncRNA Expression and Patients’ Demographical and Clinicopathological Features, Baseline Full Blood Count and Coagulation Tests

Regarding patients’ demographical and clinical features, no significant associations were found for MALAT1, TUG1 and NEAT1, regardless of the expression profile and the first treatment (*Χ*^2^, *p* > 0.05). In opposition, for MEG8, a significant association between its pre-chemotherapy levels in PBCs and KS (<2 vs. ≥2) was observed (profile A (see Section 4.7 for profile definition), *Χ*^2^, *p* = 0.009; profile B, *Χ*^2^, *p* = 0.048). When stratifying the analysis in terms of first treatment (surgery vs. chemotherapy), only those that would undergo chemotherapy as the first intervention (meaning they were treatment-naïve at the time of first sample collection) presented pre-chemotherapy MEG8 levels that were significantly associated with KS (profile A, *Χ*^2^, *p* = 0.013). In both analyses (entire cohort and subgroups), elevated pre-chemotherapy MEG8 levels were predominant in the KS < 2 group. Moreover, an association was found between XIST expression and cancer antigen 125 (CA 125) levels among those initially treated with chemotherapy. Specifically, intermediate and elevated pre-chemotherapy levels of the lncRNA were associated with high CA 125 levels (profile B, *Χ*^2^, *p* = 0.047).

Significant associations were detected for MALAT1, TUG1 and XIST regarding the baseline full blood count. For MALAT1, irrespective of the first treatment, its expression was positively associated with leukocyte (profile A, *Χ*^2^, *p* = 0.041) and neutrophil (profile A, *Χ*^2^, *p* = 0.041) counts, meaning that its high expression was linked to a higher count of these cells. The same lncRNA was also associated with haemoglobin levels, with its higher levels being predominant among those with higher haemoglobin levels (profile B, *Χ*^2^, *p* = 0.036; profile C, *Χ*^2^, *p* = 0.028). Regarding TUG1, irrespective of the first intervention, elevated neutrophil counts were linked to its higher levels (profile B; *Χ*^2^, *p* = 0.048). For XIST, a significant relationship with haemoglobin levels was found for those first treated with surgery, with its higher expression being associated with higher haemoglobin levels (profile B, *Χ*^2^, *p* = 0.033). In terms of leukocyte count, a significance was also observed (profile B, *Χ*^2^, *p* = 0.027), with a specific emphasis on the chemotherapy group (profile B, *Χ*^2^, *p* = 0.037), where higher levels of XIST were linked to a higher leukocyte count. Similarly, for neutrophil count, a significant association was identified irrespective of the treatment (profile B, *Χ*^2^, *p* = 0.027), and for the chemotherapy group (profile B, *Χ*^2^, *p* = 0.043), as lower levels of XIST were also associated with a lower neutrophil count.

Concerning the baseline coagulation tests, namely prothrombin time (PT), activated partial thromboplastin (aPTT) and international normalised ratio (INR), significant associations were detected only for MALAT1 and MEG8. Specifically, higher pre-chemotherapy MALAT1 levels were linked to lower PT times in the chemotherapy group (profile B, *Χ*^2^, *p* = 0.025). For MEG8, irrespective of the first treatment, higher levels of this lncRNA were associated with a longer PT (profile A, *Χ*^2^, *p* = 0.014; profile B and D, *Χ*^2^, *p* < 0.001). When stratified by treatment, this association remained significant for the surgery group (profile A, *Χ*^2^, *p* = 0.038) and in both chemotherapy and surgery (profile B, *Χ*^2^, *p* = 0.014 and *p* = 0.022, respectively; profile D, *Χ*^2^, *p* = 0.031 and *p* = 0.038, respectively) depending on the expression profile. A significant association was also detected between INR and MEG8 expression, regardless of the first treatment, with elevated pre-chemotherapy levels of MEG8 being more predominant among those with a higher INR (profile A and B, *Χ*^2^, *p* = 0.041 and *p* = 0.023, respectively; profile D, *Χ*^2^, *p* = 0.030). Furthermore, the lncRNA expression levels were also associated with aPTT but only in the chemotherapy group, where its elevated levels were related to a higher aPTT (profile D, *Χ*^2^, *p* = 0.044).

### 2.3. Chemotherapy Impact on lncRNA Expression

**Figure 2:**
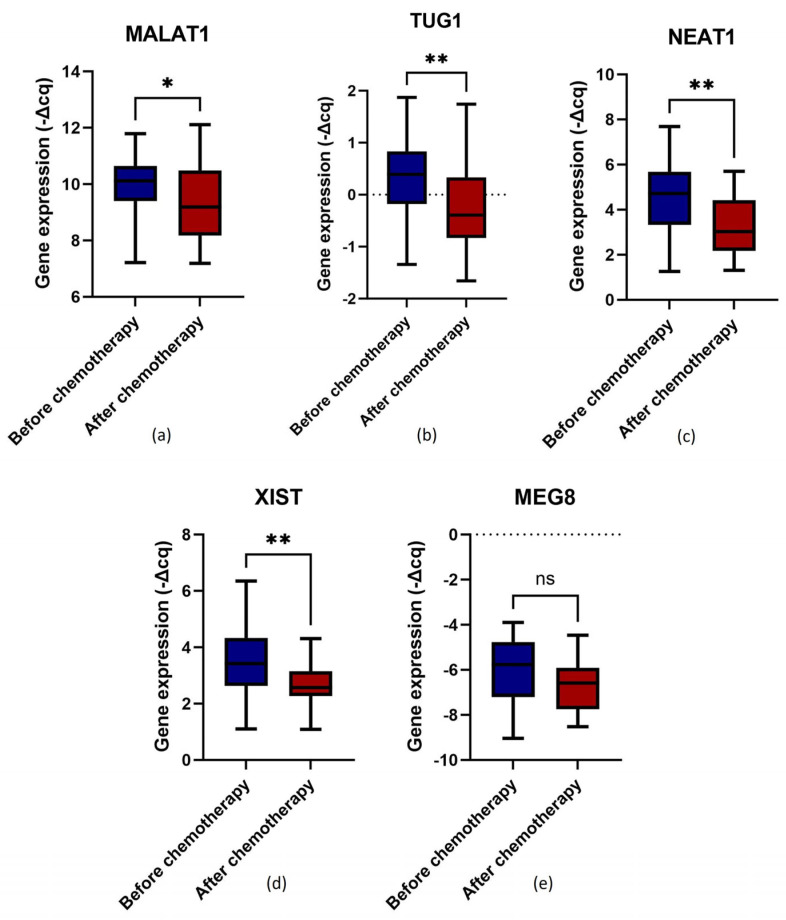
Normalised relative expression levels of lncRNAs (−∆Cq) in peripheral blood cells among ovarian cancer patients before and after the first-line chemotherapy: (**a**) MALAT1 expression; (**b**) TUG1 expression; (**c**) NEAT1 expression; (**d**) XIST expression; and (**e**) MEG8 expression; Wilcoxon matched-pairs signed rank test, * *p* < 0.05, ** *p* < 0.01; ns, non-significant.

For patients whose samples prior to and after the first-line chemotherapy were available (n = 37), the impact of chemotherapy on the expression levels of the selected lncRNAs was evaluated. Statistically significant differences were detected (Wilcoxon matched-pairs signed rank test, *p* < 0.05) between the expression levels before and after chemotherapy for MALAT1, TUG1, NEAT1 and XIST (Figure 2a (*p* = 0.047), 2b (*p* = 0.003), 2c (*p* = 0.005) and 2d (*p* = 0.009), respectively). In opposition, only a marginally significant difference was observed for MEG8 (Figure 2e, *p* = 0.058). In the stratified analyses, TUG1 levels were significantly decreased after chemotherapy in the group that underwent chemotherapy as the first intervention (n = 17; *p* = 0.029). As for those first treated with surgery (n = 20), pre-chemotherapy TUG1 and XIST expression in PBCs significantly decreased after chemotherapy (*p* = 0.035 and *p* = 0.006, respectively). For the other lncRNAs, no statistically significant differences were detected in their expression levels in the stratified analyses (Wilcoxon matched-pairs signed rank test, *p* > 0.05).

### 2.4. LncRNA Expression and OC-Related VTE Incidence

**Figure 3:**
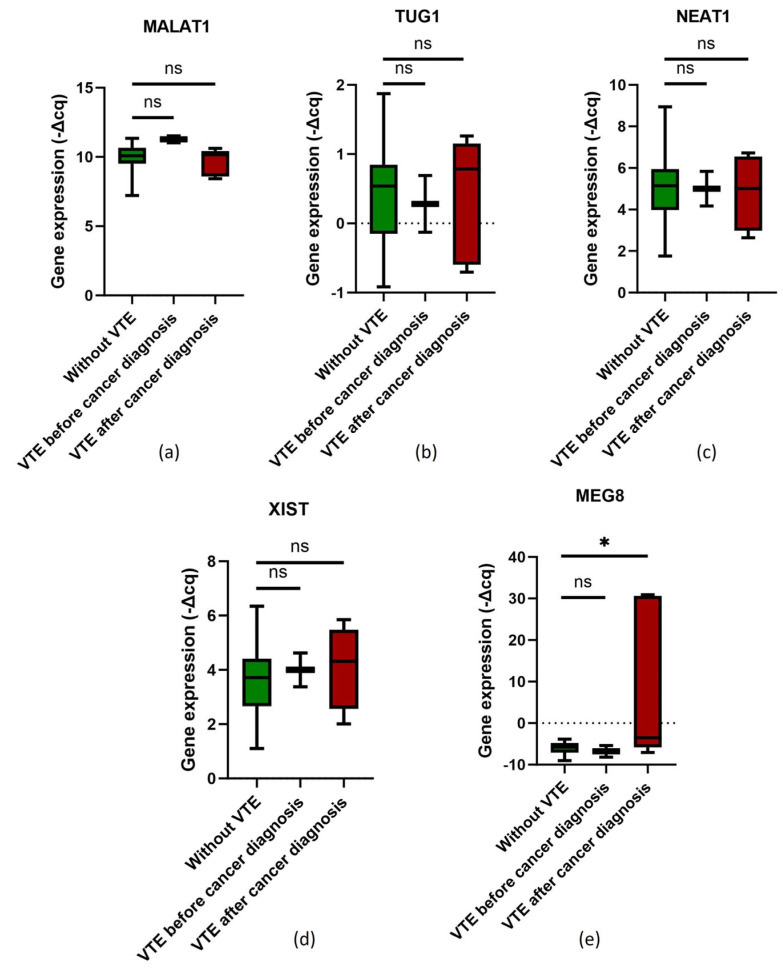
Normalised relative expression levels of lncRNAs (−∆Cq) in peripheral blood cells among ovarian cancer patients in the context of venous thromboembolism (VTE): (**a**) MALAT1 expression; (**b**) TUG1 expression; (**c**) NEAT1 expression; (**d**) XIST expression; and (**e**) MEG8 expression; Mann–Whitney *U* test, * *p* < 0.05; ns, non-significant.

The impact of the evaluated lncRNAs on OC-related VTE incidence was investigated by considering patients without VTE, those with the condition before cancer diagnosis and those with the condition after cancer diagnosis. For MALAT1, TUG1, NEAT1 and XIST, no statistically significant differences were observed between the different groups (Mann–Whitney *U* test; *p* > 0.05; Figure 3a–d), which was corroborated by the Chi-Square test (*Χ*^2^) (profiles A, B, C and D; *Χ*^2^, *p* > 0.05). Concerning MEG8, its pre-chemotherapy expression levels were significantly elevated in OC patients who later developed a VTE event after cancer diagnosis compared to those without the condition (Mann–Whitney *U* test; *p* = 0.037; Figure 3e). This result was further confirmed by the Chi-Square test (*Χ*^2^) (profile C; *Χ*^2^ test, *p* = 0.033).

### 2.5. LncRNA Expression and Patients’ Prognosis

The influence of the lncRNAs on patients’ clinical outcomes was dissected in univariable and multivariable Cox analyses adjusting for the most relevant patients’ prognostic factors. According to a multivariate analysis applying the backward Wald method, surgery (yes vs. no) and platinum sensitivity (sensitive vs. resistant) were the most relevant predictors of the risk of disease progression, while the latter had a standout performance as a predictor of the risk of death.

For MALAT1, TUG1 and XIST, no impact on the risk of OC progression and death was observed, both in univariable and multivariable Cox analyses, regardless of the expression profile (*p* > 0.05). For MEG8, no significant association with the risk of disease progression was detected in the univariable Cox analysis (*p* > 0.05). However, in a multivariable Cox analysis adjusting for surgery and platinum sensitivity, a significant impact of this lncRNA was observed. Specifically, its low pre-chemotherapy expression levels were associated with a three-fold increase in the risk of OC progression (profile A, adjusted hazard ratio (aHR) = 3.00, 95% confidence interval (95% CI), 1.06–8.51, *p* = 0.039; Table 1). As for the impact of MEG8 on the risk of death, no significant association was identified (Cox regression analysis, *p* > 0.05). Concerning NEAT1, no significant association with the risk of disease progression was detected (Cox regression analysis, *p* > 0.05). As for its impact on the risk of death, a significant association was only observed in the multivariable Cox analysis adjusting for platinum sensitivity. Specifically, its low expression levels in PBCs prior to chemotherapy were associated with a six-fold increase in the risk of death (profile D, aHR = 6.25, 95% CI, 1.60–24.43, *p* = 0.008; Table 1).

## 3. Discussion

Among other solid tumours, OC is one of the most associated with VTE [19,20,21,22,23,24,25,26]. Corroborating a two-way association between thrombosis and OC progression, increased D-dimer levels are linked to high tumour burden, chemoresistance and poor prognosis, regardless of VTE status [4,27]. Likewise, high plasma thrombin levels have been shown to negatively impact OC patients’ survival and prognosis, while elevated platelet count predicts disease recurrence [28,29]. Also, TF overexpression is linked to aggressive tumours, advanced stages and decreased survival among OC patients [30,31]. Ovary tumours are also known to stimulate platelets, endothelial and immune cells to present a procoagulant behaviour [2,32]. Together, all these players appear to act in a complex network to promote ovarian tumorigenesis allied to immunothrombosis. Concordantly, our research group previously showed that VTE-related genetic polymorphisms have a prognostic value among OC patients, regardless of VTE status [33]. Subsequently, in recent years, multiple lncRNAs have been implicated in venous thrombus formation and resolution. However, the data on cancer patients are scarce [18]. Thus, this study evaluated the impact of VTE-associated lncRNAs on the incidence of OC-related VTE and their prognostic value among OC patients.

In this cohort study, from six months before to two years after cancer diagnosis, the incidence of OC-related VTE was 23.3%, which goes in line with the current evidence, confirming that OC patients have a high pro-thrombotic risk [19,20,21,22,23,24,25,26]. Furthermore, those with VTE had worse survival compared to their counterparts, particularly when focusing on patients with an event after OC diagnosis (log-rank test, *p* = 0.028), which corroborates the existing evidence [34,35,36]. As for PFS, no significant association was detected. Given the negative effect of VTE, it is imperative to delve into more effective thromboprophylaxis measures. Until now, the most studied score for predicting CAT is KS [37,38,39,40]. In this study, the score had a poor performance, unable to predict OC-related VTE onset, which was also previously described [25]. Although additional research in larger cohorts is needed, these preliminary findings reinforce the need for better and more tailored VTE predictive models.

Regarding the evaluated lncRNAs, starting with MALAT1, it has been pointed out as a major player in thrombogenesis due to its downregulation in atherosclerotic plaques and its association with endothelial function [41,42]. Inconsistent results, however, led to the hypothesis that this lncRNA may have a cell- and/or context-dependent role in VTE [18,42,43,44]. In the present study, higher pre-chemotherapy expression levels of MALAT1 in PBCs, irrespective of the first treatment (surgery vs. chemotherapy), were associated with higher leukocyte (profile A, *Χ*^2^, *p* = 0.041) and neutrophil (profile A, *Χ*^2^, *p* = 0.041) counts. This suggests that MALAT1 expression in PBCs is attributed, at least partially, to neutrophils. Indeed, as previously mentioned, neutrophils are implicated in immunothrombosis through the release of NETs (NETosis), which is promoted, for instance, by TF and platelets to increase the pro-thrombotic cascade [9]. Concordantly, exosomal MALAT1 from human umbilical vein endothelial cells (HUVECs) was previously revealed to stimulate NETosis, triggering a proinflammatory response [45]. The data hint that under pro-inflammatory and pro-thrombotic settings (e.g., tumour microenvironment), endogenous or exosomal MALAT1 may promote neutrophil activation, favouring thrombus formation [42,43]. In line with the role of neutrophils in MALAT1 expression, in this study, the lncRNA expression levels were significantly decreased after chemotherapy (Wilcoxon matched-pairs signed rank test, *p* = 0.047). Indeed, by lessening the tumour burden, chemotherapy might have diminished the cancer stimulatory effect on the PBCs [46]. Moreover, the therapy itself is known to negatively impact leukocyte activity and counts, causing conditions such as neutropenia and leukopenia [47,48]. Also, chemotherapy can decrease platelet counts and cause endothelial dysfunction, altering the signal from these haemostatic key players to leukocytes (i.e., immunothrombosis) [9,49,50]. Additionally, higher MALAT1 levels were found to be associated with higher haemoglobin levels (profile B, *Χ*^2^, *p* = 0.036; profile C, *Χ*^2^, *p* = 0.028). The current evidence on the link between MALAT1 and haemoglobin is, however, scarce and conflicting [51,52]. Furthermore, in the present study, pre-chemotherapy expression levels of MALAT1 in PBCs were also found to be associated with lower PT in the group that had chemotherapy as the first treatment (profile B, *Χ*^2^, *p* = 0.025). Although the coagulation tests did not present a predictive ability concerning VTE incidence (most likely due to the low number of patients with abnormal PT values), this finding suggests that, at OC diagnosis, MALAT1 expression in PBCs might be linked to haemostatic abnormalities, particularly in the extrinsic coagulation pathway [47,48,53,54]. However, no significant association with OC-related VTE was found, possibly due to the context-dependent role of this lncRNA and the limited cohort size. As for its implications in OC pathways, MALAT1 is usually upregulated, which is associated with advanced disease stages, high tumour burden and a poor prognosis [55,56,57,58,59]. However, no significant associations of MALAT1 with OS and PFS were detected in this study. Further investigation with larger cohorts is necessary to assess the implications of this lncRNA in PBCs among OC patients.

Regarding TUG1, by serving as a decoy, this lncRNA seems to exert a protective effect against DVT [60]. In this study, elevated pre-chemotherapy levels of TUG1 in PBCs, irrespective of the primary treatment, were linked to higher neutrophil counts (profile B; *Χ*^2^, *p* = 0.048). Indeed, TUG1 has been reported to directly facilitate the transcription of leucine-rich alpha-2-glycoprotein 1 (LRG1), a modulator of angiogenesis and inflammation, constitutively expressed by neutrophils. Interestingly, the little existing data indicate that LRG1 promotes the extravasation and activation of neutrophils, stimulating NETosis, which, as mentioned before, is implicated in immunothrombosis [61,62]. Furthermore, similar to MALAT1, TUG1 expression levels in PBCs decrease after the first-line chemotherapy (Wilcoxon matched-pairs signed rank test, *p* = 0.003), most likely due to the impact of the therapy on cancer burden, leukocytes (and neutrophils specifically), platelets and endothelial cells, overall leading to a lower TUG1 expression. Regarding the baseline haemoglobin levels and the coagulation tests, no significant associations involving TUG1 were observed. The lncRNA also did not present a significant impact on VTE incidence among the OC patients. As for its implications in OC pathways regardless of VTE, the roles of this lncRNA also seem to be conflicting or context-dependent [63,64,65,66]. In the present study, TUG1 did not show any impact on the risk of disease progression or death. Given the reported roles of TUG1 in VTE and OC, further studies are warranted.

Serum levels of the lncRNA NEAT1 were previously found to be upregulated in DVT patients compared to healthy individuals. In HUVECs, this lncRNA is implicated in endothelial dysfunction and coagulation/fibrinolysis imbalances [67,68]. This study found no statistically significant associations between pre-chemotherapy NEAT1 expression levels in PBCs and the baseline full blood count. According to the literature, this lncRNA regulates immune cell functions and, although with a low immune cell specificity, NEAT1 seems to be mainly expressed by monocytes and lymphocytes [69,70]. Indeed, while cell counts can impact the expression levels of a gene within a sample, gene expression activity is often the primary contributor, which could explain the absent association between NEAT1 levels and leukocyte counts [71]. Furthermore, decreased levels of this lncRNA were observed after the first-line chemotherapy (Wilcoxon matched-pairs signed rank test, *p* = 0.005), which could be once again explained by the negative impact of chemotherapy on tumour burden, leukocytes, platelets and endothelial cells. As for its roles in thrombogenesis, NEAT1 did not present a significant association with the baseline coagulation tests or OC-related VTE incidence. As previously mentioned, the study that linked NEAT1 to VTE was conducted by analysing the expression of the lncRNA in serum and HUVECs [67]. Additional investigation with a larger cohort size is needed to unravel the role of NEAT1 expression in PBCs in thrombogenesis among OC patients. Concerning ovarian tumorigenesis, a previous study demonstrated that NEAT1 was upregulated in OC compared to non-neoplastic tissues. Also, the lncRNA expression levels were positively associated with the International Federation of Gynecology and Obstetrics (FIGO) stage and tumour grade, being an independent prognostic factor of OC [68]. However, in the present study, diminished pre-chemotherapy NEAT1 expression levels in PBCs were significantly associated with an increased risk of mortality adjusting for platinum sensitivity (profile D, aHR = 6.25, 95% CI, 1.60–24.43, *p* = 0.008). Curiously, NEAT1 low levels were previously reported to lead to an excessive accumulation of promyelocytes, potentially leading to coagulation abnormalities [72,73,74]. Also, in OC, NEAT1-enriched extracellular vesicles derived from M2-polarised tumour-associated macrophages have been previously demonstrated to favour immune evasion [75]. Whether these two seemingly isolated mechanisms are interrelated and underlying the negative effect of low NEAT1 expression in PBCs needs to be explored. No significant association between the lncRNA expression and PFS was detected. Overall, more studies with larger cohort sizes are required to establish the role of NEAT1 in VTE and OC.

The lncRNA XIST was previously found to be highly expressed in the plasma of DVT patients, possibly acting as a decoy by sponging miR-103a-3p, which in turn, targets high-mobility group box 1 (HMGB1), a pro-thrombotic protein that promotes the activation and aggregation of platelets [76,77]. In the present study, intermediate and higher pre-chemotherapy expression levels of XIST in PBCs were associated with elevated leukocyte (profile B, *Χ*^2^, *p* = 0.027) and neutrophil (profile B, *Χ*^2^, *p* = 0.027) counts, with a specific emphasis on those that were first treated with chemotherapy (profile B, *Χ*^2^, *p* = 0.037 and profile B, *Χ*^2^, *p* = 0.043, respectively). Like MALAT1 and TUG1, the results suggest that the expression of XIST in PBCs was attributed, at least partially, to neutrophil counts. Concordantly, this lncRNA has been previously implicated in neutrophil-associated biological processes, including NETosis [78,79]. Concerning chemotherapy’s impact, XIST expression levels were significantly decreased after the first-line chemotherapy (Wilcoxon matched-pairs signed rank test, *p* = 0.009). Furthermore, a significant relationship with haemoglobin levels was found in the surgery group, with intermediate and elevated levels of XIST linked to higher haemoglobin levels (profile B, *Χ*^2^, *p* = 0.033). However, the existing data on this association are scarce. Regarding haemostatic abnormalities assessed by baseline coagulation tests and the incidence of OC-related VTE, no significant associations with XIST were detected. Nevertheless, the existing evidence on its implications in VTE pathways addresses the expression of the lncRNA in plasma [79]. Thus, additional investigation with a larger cohort size is needed to unravel the role of PBCs’ expression of XIST in thrombogenesis. Concerning OC progression, there are contradictory findings surrounding this lncRNA. A study showed that XIST is highly expressed in epithelial OC (EOC) tissues and cell lines, being strongly associated with tumour grade, FIGO stage and distant metastasis, and representing an independent prognostic factor [79,80,81]. In opposition, its overexpression has been associated with longer survival in OC patients, being downregulated in advanced stages, compared to early disease stages [82]. In the present study, no impact of XIST on the risk of disease progression or death was found. However, within the chemotherapy group, intermediate and elevated levels of the lncRNA were associated with elevated levels of serum CA 125 (profile B, *Χ*^2^, *p* = 0.047). As this marker is known to be positively correlated with OC burden, XIST might also increase with tumour progression [83]. However, as mentioned before, the available literature has conflicting findings regarding the role of XIST in ovarian tumorigenesis, and the implications of PBCs’ expression of this lncRNA in OC progression are yet unclear. Thus, further studies are needed.

The lncRNA MEG8 is dysregulated in several disorders, such as lung, ovarian and colorectal cancers, as well as gestational diabetes mellitus, diabetic nephropathy and ischemic heart disease [84,85]. In this study, MEG8 expression levels in PBCs were not associated with the baseline full blood count, nor impacted by the first-line chemotherapy. This suggests that the expression level of this lncRNA is mainly attributed to the gene expression activity in PBCs, which is sustained even with chemotherapy. Furthermore, elevated pre-chemotherapy MEG8 levels were significantly associated with lower KS (≥2 vs. <2), irrespective of the first treatment (profile A, *Χ*^2^, *p* = 0.009; profile B, *Χ*^2^, *p* = 0.048) and among those that had chemotherapy as a first intervention (profile A, *Χ*^2^, *p* = 0.013). In opposition, the lncRNA expression was not individually associated with either of the KS parameters (pre-chemotherapy body mass index (BMI), haemoglobin levels and platelet and leukocyte counts) [40]. Also, elevated MEG8 levels were associated with prolonged PT, aPTT and INR, which indicates its potential association with abnormalities of the haemostatic system, involving both the intrinsic and extrinsic pathways [53,54]. Concordantly, the lncRNA was significantly elevated among OC patients who later experienced a VTE event compared to those who remained VTE-free (Mann–Whitney *U* test, *p* = 0.037; *Χ*^2^ test, *p* = 0.033). As mentioned, elevated MEG8 levels were also significantly associated with lower KS. This seemly conflicting result can be attributed to the poor performance of KS in predicting OC-related VTE incidence. Inclusively, among OC patients, those classified with low VTE risk (KS < 3 or KS < 2, depending on the cut off) often end up suffering from the condition, according to different studies [39]. In contrast with the other evaluated lncRNAs, MEG8 has not yet been directly linked to VTE in previous studies. Instead, it is reported to be a scaffold lncRNA repressing the expression of tissue factor pathway inhibitor 2 (TFPI2) [18,85]. This haemostatic protein is expressed in cells such as monocytes or macrophages and endothelial cells, playing dual roles: as an anticoagulant by targeting the extrinsic coagulation pathway (also known as the TF coagulation pathway) and as an anti-fibrinolytic protein by inhibiting plasmin. Collectively, TFPI2 seems to play a context-dependent role in VTE pathways, influencing both thrombus formation and its resolution [86,87,88,89]. Therefore, as MEG8 downregulates the expression of TFPI2, which is also expressed among leukocytes, the present study results suggest that TFPI2 as an anticoagulant protein, rather than an anti-fibrinolytic protein, might be the primary mechanism underlying the promoting effect of MEG8 in blood clot formation. As such, due to the MEG8 inhibition of TFPI2 expression, the active TF coagulation pathway contributes to OC-related VTE occurrence. In accordance with its context-dependent role, elevated serum TFPI2 levels were reported to be a diagnostic marker of VTE among OC patients [90,91]. This hints that, in opposition to blood clot formation, MEG8 levels may be downregulated at thrombus resolution, leading to TFPI2 upregulation, inhibiting plasmin and impairing thrombus resolution. In sum, elevated pre-chemotherapy MEG8 levels in PBCs could be a predictor of a later OC-related VTE occurrence. As for the role of this lncRNA in OC progression, in the present study, lower MEG8 expression levels before chemotherapy were linked to a three-fold increase in the risk of disease progression adjusted for surgery and platinum sensitivity (profile A, aHR = 3.00, *p* = 0.039). In accordance with this, a study utilising integrated bioinformatics analysis demonstrated that diminished MEG8 levels in OC tissues were associated with a poorer prognosis [92]. Interestingly, in opposition to other tumours, TFPI2 is often overexpressed in ovarian clear cell carcinoma (the EOC subtype most associated with VTE), though its precise role and the underlying mechanisms remain unclear [93,94,95,96]. Furthermore, low MEG8 levels in endothelial cells are known to impair endothelial function, which in turn, in addition to promoting VTE, also plays a role in cancer by amplifying the tumour inflammatory signalling and consequently promoting tumorigenesis [85,97,98]. As for the implications of MEG8 expression in PBCs in ovarian tumorigenesis, the data are scarce. Nevertheless, MEG8 has been previously linked to immune infiltration in OC [92]. Additionally, its lower levels may lead to an overexpression of TFPI2, which, as previously mentioned, has an inhibitory activity towards plasmin [86]. In turn, this pro-fibrinolytic protein is also implicated in tumour growth and dissemination via extracellular matrix degradation, influencing tumour metastasis [99]. Of note, no significant association between MEG8 expression and OS was detected. Further studies with larger cohorts are needed for clarification.

Despite the promising results, the present study had limitations that need to be acknowledged. Firstly, the main constraint was the small cohort size, which may have lowered this study’s statistical power, potentially impairing the generalisation of the findings to a broader population. Furthermore, the retrospective design of this study impeded the gathering of other relevant data. For instance, it would be important to assess the lncRNA expression close to VTE occurrence to evaluate the diagnostic capability of these non-coding RNAs. Also, VTE was not actively screened, which may have led to a potential underestimation of asymptomatic VTE events. This oversight may have inclusively contributed to the poor performance of KS. Moreover, there was an absence of control groups comprising healthy participants and VTE patients without cancer, which hindered the comparison of the lncRNA expression in health and disease contexts.

Regarding the strengths of this study, firstly, the population under evaluation was relatively homogeneous, which enhances the internal validity of our findings. Second, all the major risk factors related to VTE occurrence were accounted for. Moreover, this study was focused on deregulated lncRNAs in PBNCs, demonstrating the potential clinical relevance of liquid biopsies in OC prognosis. Overall, this study represents a pioneering effort in shedding light on the unexplored territory of VTE-related lncRNAs within the context of CAT, since, up until now, no studies have been conducted on the role of these lncRNAs among cancer patients.

## 4. Materials and Methods

### 4.1. Population Description

A retrospective cohort study was performed with adult European-descent patients from the North Region of Portugal with a confirmed diagnosis of EOC, admitted from March 2017 to April 2022 to the Clinic of Gynaecology of the Portuguese Oncology Institute of Porto (IPO Porto) for first-line treatment. In this study, patients were excluded if they (1) had other malignant diseases prior to or after OC diagnosis; (2) were pregnant or breastfeeding at cancer diagnosis; (3) had autoimmune diseases and/or were under immunosuppressive treatments; (4) had an acute infection at cancer diagnosis; (5) were under anticoagulation due to other diseases rather than VTE; and (6) had factor V Leiden and prothrombin G20210A polymorphisms. Therefore, 40 OC patients were enrolled, for whom biological material was available before the first line of platinum-based chemotherapy was available. Most patients had serous (33 (82.5%)), followed by clear-cell carcinoma (3 (7.5%)), carcinosarcoma (2 (5%)), endometrioid (1 (2.5%)) and mixed EOC (1 (2.5%)).

Disease staging was conducted according to the FIGO system updated in 2021 [100]. Data concerning the patients’ demographical and clinicopathological features (including VTE history), baseline full blood count and coagulation tests were obtained from their medical data files. CAT was defined as an event occurring six months before to two years after cancer diagnosis [101]. It is worth noting that no active screening was conducted since this measure is not yet incorporated into the clinical routine procedures of IPO Porto. The median follow-up time was 24.5 months (minimum = 9.0 months; maximum = 76.0 months). A description of the study cohort characteristics is given in Table 2.

### 4.2. LncRNA Selection

Based on a literature review [18], lncRNAs were selected if they (1) had involvement in VTE; (2) had an association with haemostatic key players; (3) were expressed by PBCs, particularly monocytes, neutrophils and platelets; and (4) had available TaqMan assays for gene expression analysis. Additionally, those with known prognostic value in cancer were prioritised. Thus, the investigation was based around the subsequent lncRNAs: metastasis-associated lung adenocarcinoma transcript 1 (MALAT1), taurine-upregulated gene 1 (TUG1), nuclear-enriched abundant transcript 1 (NEAT1; also known as nuclear paraspeckle assembly transcript 1), X-inactive-specific transcript (XIST) and maternally expressed 8 (MEG8).

### 4.3. Sample Collection and Processing

Peripheral blood samples were collected for each patient in ethylenediamine tetraacetic acid (EDTA)-coated tubes using a standard venipuncture technique. Samples were gathered before and, whenever possible, after the first-line chemotherapy. PBCs were isolated by first lysing the erythrocytes using an ammonium-chloride–potassium (ACK) solution (1×). The samples were then frozen at −20 °C for 20 min and then centrifugated for 10 min at 2500 rpm at room temperature. After discarding the supernatant, successive washes with ACK solution (1×) and phosphate-buffered saline (PBS) solution (1×) were conducted, followed by rounds of centrifugation for 10 min at 2500 rpm at room temperature to separate and discard the supernatant. The obtained pellet of PBCs (without erythrocytes) was further diluted and conserved in TriPure^®^ Isolation Reagent (Roche Applied Science, Penzberg, Germany). Samples of PBCs were stored at −80 °C until further use.

### 4.4. Total RNA Extraction

Total RNA from PBC samples was extracted using the GRS RNA kit—Blood & cultured cells (#GK08.0100) (Grisp Research Resolutions^®^, Porto, Portugal) following the manufacturer’s instructions with an adjustment (the duration of the DNAse treatment (DNase I set (#GKC01.0100) was extended). RNA concentration and purity were analysed using the NanoDrop Lite spectrophotometer (Thermo Scientific^®^, Waltham, MA, USA). Following extraction, RNA samples were stored at −80 °C until use.

### 4.5. cDNA Synthesis

RNA samples (150 ng) were used as templates to generate complementary DNA (cDNA) using the High-Capacity cDNA Reverse Transcription Kit (Applied Biosystems^®^, Carlsbad, CA, USA) following the manufacturer’s instructions. The conversion was conducted in a Mycycler^TM^ Thermal cycler (Bio-Rad Laboratories, Hercules, CA, USA) with the recommended cycle conditions: 10 min at 25 °C, 120 min at 37 °C and 5 min at 85 °C. Negative controls (lacking RNA) were incorporated in all reactions.

### 4.6. Relative Quantification of lncRNAs

The expression levels of the lncRNAs were quantified through quantitative real-time polymerase chain reaction (qRT-PCR) using a StepOnePlus^TM^ qPCR system (Applied Biosystems^®^, Foster City, CA, USA). Data analysis was carried out using the StepOne software (version 2.3, Applied Biosystems^®^, Foster City, CA, USA). Each PCR reaction was performed using 5 µL of 2× TaqMan^TM^ Gene Expression Master mix (Applied Biosystems^®^, Foster City, CA, USA); 3.5 µL of nuclease-free water; 0.5 µL of 20× TaqMan^TM^ Gene Expression Assays (Applied Biosystems^®^, Foster City, CA, USA) for *MALAT1* (Hs00273907_s1), *TUG1* (Hs00215501_m1), *XIST* (Hs01079824_m1), *NEAT1* (Hs01008264_s1), *MEG8* (Hs00419701_m1), *GAPDH* (Hs99999905_m1) and *HPRT1* (Hs02800695_m1); and 1.0 µL of cDNA sample, making a total volume of 10 µL. For all reactions, the thermal cycling conditions were: 2 min at 50 °C, 10 min at 95 °C, 45 cycles of 15 s at 95 °C and 1 min at 60 °C. The quantification was conducted in triplicate and negative controls (without cDNA) were included in all reactions. All targets and endogenous controls for each sample were amplified in the same plate. Standard deviations (SDs) of cycle threshold (Ct) values superior to 0.5 were dismissed. The same baseline and threshold were set for each plate using the analysis software for qRT-PCR from the Thermo Fisher Connect platform (Thermo Fisher Scientific, Waltham, MA, USA) to generate Ct values for all the targets and endogenous controls in each sample. *GAPDH* and *HPRT1* were tested as endogenous controls.

### 4.7. Statistical Analysis

Data analysis was conducted using IBM SPSS Statistics for Windows (version 29, IBM Corp., Armonk, NY, USA) and GraphPad Prism software (version 10.0, GraphPad Software Inc., La Jolla, CA, USA).

The lncRNAs’ normalised relative expression was determined using the Livak method. Among the tested endogenous controls, *HPRT1* was used to normalise the lncRNAs’ expression levels given its more stable expression (i.e., lowest SD values) compared to *GAPDH*. The interquartile range (IQR) method was applied to identify outliers in the normalised relative expression levels of the lncRNAs, which were further dismissed. 

For each lncRNA, four profiles of expression were defined: profile A (low vs. high expression based on the median value of the normalised relative expression levels), profile B (low vs. intermediate vs. high expression dictated by terciles), profile C (low vs. high expression with the first and second tercile regarded as low expression and the third tercile as a high expression) and profile D (low vs. high expression with the former encompassing the first tercile and the latter the second and third terciles). All the profiles were evaluated in each analysis employing the lncRNAs’ normalised relative expression levels as a categorical variable.

Associations of VTE incidence and the pre-chemotherapy levels of the lncRNAs with patient’s demographic and clinicopathological factors (Table 2), baseline full blood count and coagulation tests (Table 3) were assessed using the Chi-Square test (*Χ*^2^), dismissing those with VTE before cancer diagnosis.

Depending on the data distribution according to the Shapiro–Wilk test, the Wilcoxon matched-pairs signed rank test or paired t-test was employed to evaluate the impact of first-line chemotherapy on the expression levels of the lncRNAs in PBCs. This analysis considered only patients with samples before and after the first-line chemotherapy. 

The statistical differences in the pre-chemotherapy expression levels of the lncRNAs according to OC-related VTE occurrence were assessed using either Student’s *t*-test or Mann–Whitney *U* test (for normal and non-normal distribution, respectively). For confirmation purposes, the Chi-Square test (*Χ*^2^) was also employed. Alive patients were characterised as VTE-free only if their follow-up time was at least 2 years without evidence of thrombotic events. Thus, those with insufficient follow-up time were dismissed in analyses concerning VTE incidence.

To evaluate the impact of VTE and the lncRNAs on patients’ prognosis, clinical outcome measures included PFS and OS. The former represents the interval between treatment initiation and either the date of first disease progression or recurrence, related mortality, or the patient’s last clinical evaluation. The latter was defined as the time from OC diagnosis until death due to all causes or last evaluation. The endpoint definition was made according to Response Evaluation Criteria in Solid Tumours (RECIST) criteria updated in 2009 (RECIST 1.1). The Kaplan–Meier method was employed to generate survival curves, while survival probabilities were analysed via the log-rank test. The impact of the evaluated lncRNAs on the risk of disease progression and death was assessed using the Cox proportional-hazards model, which was adjusted for the most relevant factors previously identified by applying the backward Wald method. In these analyses, those with VTE before OC diagnosis were dismissed.

Of note, in opposition to those first submitted to surgery, those that were first treated with chemotherapy (neoadjuvant chemotherapy or only chemotherapy in the disease management) were treatment-naïve at the time of sample collection. Thus, subgroup analyses based on the first treatment (surgery vs. chemotherapy) were conducted in all statistical assessments implicating the lncRNA-normalised relative expression levels. 

All tests conducted were two-sided and a 5% level of significance was established.

## 5. Conclusions

OC stands out as the most lethal gynaecological malignancy. This condition has a close relationship with VTE, in which tumour cells hijack the haemostatic system to drive cancer progression [102]. Recently, lncRNAs have been implicated in VTE pathogenesis but their roles among cancer patients are yet to be explored [18]. As they might constitute valuable biomarkers for both CAT prediction and OC management, the impact of five lncRNAs, including MALAT1, TUG1, NEAT1, XIST and MEG8, was evaluated in a cohort of 40 OC patients. According to the findings, these patients have a high pro-thrombotic risk and the VTE negatively impacts their survival. Among the evaluated lncRNAs, MEG8 in PBCs was a predictor of OC-related VTE, with its association with the baseline coagulation tests reflecting a possible role of this lncRNA in haemostatic abnormalities. Thus, MEG8 might hold the potential to be an attractive tool to predict VTE risk among OC patients using liquid biopsies, thus offering valuable insights into a patient’s thromboembolic risk profile. Interestingly, in line with the bilateral relationship between tumour cells and the haemostatic system, MEG8 was also demonstrated to impact the risk of OC progression regardless of VTE. Moreover, NEAT1 in PBCs was associated with the risk of death, emerging as a potential OC prognostic biomarker. While these preliminary findings are promising, they should be addressed carefully given the small cohort size. Further validation studies in larger cohorts are needed, preferably using a multicentric approach for more robust data. To our current understanding, this investigation is the first to propose lncRNAs in PBCs as potential predictors of OC-related VTE. These RNAs might enable a more focused screening and justifiable thromboprophylaxis for those at high risk, thereby safely reducing the occurrence of VTE events without adversities (e.g., bleeding disorders). Likewise, they could assess the prognosis among these patients in a non-invasive manner, helping tailor treatment strategies to improve their clinical outcomes. Further, performing single-cell RNA sequencing would be important to better understand which individual PBCs are responsible for expressing these lncRNAs. Additionally, it would be imperative to include control groups comprising healthy individuals and VTE subjects without cancer. Beyond PBCs, the expression of these lncRNAs in OC and normal ovarian tissues is important to offer deeper insights into their roles in different pathophysiological settings. Furthermore, functional studies should be conducted to dissect the biological processes underlying the role of these lncRNAs in OC-related VTE and OC progression, including their target proteins. In sum, more studies in this field and with more real-world OC patients are required to clarify the clinical use of lncRNAs as OC biomarkers in an era of liquid biopsies.

## Figures and Tables

**Table 1:** Multivariable Cox regression analysis on the risk of disease progression (n = 29) and risk of death among OC patients (n = 35) according to pre-chemotherapy levels of MEG8 and NEAT1 in PBCs, respectively.

Variable	aHR	95% CI	*p*-Value	Event
MEG8 (Lower vs. higher expression ^1^)	3.00	1.06–8.51	0.039	Risk of disease progression
Surgery (Yes vs. no ^1^)	0.003	0.00–0.04	<0.001	
Platinum sensitivity (Sensitive vs. resistant ^1^)	0.01	0.00–0.14	<0.001	
NEAT1 (Lower vs. higher expression ^1^)	6.25	1.60–24.43	0.008	Risk of death
Platinum sensitivity (Sensitive vs. resistant ^1^)	0.10	0.03–0.33	<0.001	

^1^ Reference group. Abbreviations: aHR, adjusted hazard ratio; CI, confidence interval; OC, ovarian cancer; PBCs, peripheral blood cells.

**Table 2:** Demographic and clinicopathological characterisation of OC patients (n = 40).

Variable	n (%)
Age (years) *	63.1 ± 12.5
<63	19 (47.5)
≥63	21 (52.5)
**BMI (kg/m^2^)**	
<26	19 (47.5)
≥26	20 (50.0)
**Hormonal status**	
Premenopausal	10 (25.0)
Postmenopausal	30 (75.0)
**ECOG**	
≤1	36 (90.0)
>1	4 (10.0)
**Inherited mutations linked to ovarian cancer**	
*BRCA1/2*	4 (10.0)
Other	1 (2.5)
None	35 (87.5)
**Histological grade** **^†^**	
Low	1 (2.5)
High	38 (95.0)
**FIGO stage ^†^**	
I/II	10 (25.0)
III/IV	30 (75.0)
**Serum CA 125 levels (U/mL)**	
<1195	19 (47.5)
≥1195	20 (50.0)
**First-line treatment**	
Surgery plus adjuvant chemotherapy	21 (52.5)
Neoadjuvant chemotherapy plus surgery	1 (2.5)
Neoadjuvant chemotherapy plus surgery plus adjuvant chemotherapy	9 (22.5)
Chemotherapy only	9 (22.5)
**Platinum sensitivity ^‡^**	29 (72.5)
**Maintenance therapy**	
PARP inhibitors	13 (32.5)
Bevacizumab	4 (10.0)
**Anticoagulation therapy ^§^**	2 (5.0)
**Platelet anti-aggregation therapy**	7 (17.5)
**KS**	
<2	21 (52.5)
≥2	15 (37.5)

* Presented as mean ± standard deviation. ^†^ Defined following the FIGO Cancer Report 2021 [100]. ^‡^ A platinum-sensitive patient was considered as having cancer progression after at least six months after the last dose of platinum-based chemotherapy. ^§^ Patients who had cancer-related venous thromboembolism prior to OC diagnosis. Except for first-line treatment, platinum sensitivity and maintenance therapy, all the factors were defined at cancer diagnosis. For patients’ age, categories of the variable were defined based on the mean value given its normal distribution (Shapiro–Wilk test, *p* = 0.738), whereas for BMI and serum CA 125 levels, the median value was used as a cut off since the variables were non-normally distributed (Shapiro–Wilk test, *p* < 0.05). For some patients, there was missing information, namely one for BMI, histological grade and serum CA 125 levels and four for KS. Abbreviations: BMI, body mass index; BRCA, Breast Cancer Gene; CA 125, cancer antigen 125; ECOG; Eastern Cooperative Oncology Group; FIGO, International Federation of Gynecology and Obstetrics; KS, Khorana score; OC, ovarian cancer; PARP, Poly (ADP-ribose) polymerase.

**Table 3:** Baseline full blood count and coagulation tests of OC patients (n = 40).

Baseline Full Blood Count		Baseline Coagulation Tests	
Variable *	n (%)	Variable *	n (%)
**Haemoglobin levels (U/mL)**		**PT (s)**	
<12.5	19 (47.5)	<14.3	17 (42.5)
≥12.5	18 (45.0)	≥14.3	18 (45.0)
**Platelet count (×10^9^/L)**		**aPTT (s)**	
<288	19 (47.5)	<26.9	17 (42.5)
≥288	18 (45.0)	≥26.9	17 (42.5)
**Leukocyte count (×10^9^/L)**		**INR**	
<28.2	19 (47.5)	<1.1	17 (42.5)
≥28.2	18 (45.0)	≥1.1	17 (42.5)
**Neutrophil count (×10^9^/L)**			
<5.1	19 (47.5)		
≥5.1	18 (45.0)		
**Monocyte count (×10^9^/L)**			
<0.6	17 (42.5)		
≥0.6	18 (45.0)		
**Lymphocyte count (×10^9^/L)**			
<1.6	17 (42.5)		
≥1.6	18 (45.0)		

* The categories were defined based on the median value since the variables were non-normally distributed (Shapiro–Wilk test, *p* < 0.05). For some patients, there was missing information, namely three for haemoglobin levels, platelet, leukocyte and neutrophil counts, five for monocyte and lymphocyte counts and PT, and six for aPTT and INR. Abbreviations: aPTT, activated partial thromboplastin; INR, international normalised ratio; OC, ovarian cancer; PT, prothrombin time.

## Data Availability

The data presented in this study are available on request from the corresponding author. The data are not publicly available, due to the privacy of participating patients and the hospital.
